# The ubiquitin–proteasome pathway protects *Chlamydomonas reinhardtii* against selenite toxicity, but is impaired as reactive oxygen species accumulate

**DOI:** 10.1093/aobpla/plu062

**Published:** 2014-10-08

**Authors:** Patrick Vallentine, Chiu-Yueh Hung, Jiahua Xie, Doug Van Hoewyk

**Affiliations:** 1 Department of Biology, Coastal Carolina University, Conway, SC 29526, USA; 2 Department of Pharmaceutical Sciences, BRITE Institute, North Carolina Central University, Durham, NC 27707, USA

**Keywords:** *Chlamydomonas*, malformed selenoprotein, mitochondrial superoxide, proteasome, reactive oxygen species, selenium, ubiquitin

## Abstract

The ubiquitin–proteasome pathway (UPP) coordinates a myriad of physiological processes in higher plants, including abiotic stress responses, but it is less well characterized in algal species. In this study, the green alga *Chlamydomonas reinhardtii* was used to gain insights into the role of the UPP during moderate and severe selenite stress at three different time points. The data indicate that activity of the UPP in response to selenium (Se) stress was both time and dose dependent. Moderate selenite stress increased proteasome activity, protein ubiquitination and the proteasomal removal of malformed selenoproteins. However, severe Se stress caused by prolonged selenite treatment or high selenite concentration decreased proteasome activity, inhibited protein ubiquitination and prevented the proteasomal removal of selenoproteins. The UPP impairment during severe Se stress was associated with the observed accumulation of reactive oxygen species (ROS), including mitochondrial superoxide. Additionally, proteasomal inhibition decreased the concentration of chlorophyll in cultures challenged with Se. Therefore, although the UPP protects *Chlamydomonas* against Se stress, severe oxidative stress induced by selenite toxicity likely hinders the UPP's capacity to mediate a stress response. The possibility that stress tolerance in plants is dependent upon optimal UPP activity and maintenance is discussed.

## Abstract (precis)

Plants subjected to stress imposed by their environment often accumulate misfolded proteins, that would be cytotoxic if not properly removed by the ubiquitin-proteasome pathway (UPP). During severe stress, the UPP becomes impaired, but the mechanisms that damage it are not well understood in plants. In this study, the effects of mild and severe stress selenium on the UPP were analyzed using the unicellular green algae *Chlamydomonas*. Mild selenium stress increased proteasome activity. However, inhibition of the UPP caused by severe selenium stress was associated with reactive oxygen species, including mitochondrial superoxide. Additionally, this is the first time that proteasome activity has been reported in lower plants.

## Introduction

Plants are sessile organisms that inevitably must confront various types of stress in their environment. A signature of abiotic stress in plants is the accumulation of reactive oxygen species (ROS), which have the capacity to induce lipid peroxidation, break or mutagenize DNA and damage proteins (Apel and Hirt 2004). Reactive oxygen species can directly damage proteins by oxidizing amino acid residues or promoting protein unfolding (Buchberger *et al.* 2010). Minimizing the accumulation of oxidized and misfolded proteins during abiotic stress is essential in order to prevent the accumulation of protein aggregates that would otherwise result in impaired cellular homoeostasis and ultimately necrosis. This can be achieved by either repairing damaged proteins via chaperone-mediated processes or targeting irreparable proteins for proteolysis via the lysosome or the ubiquitin–proteasome pathway (UPP) (Liu and Howell 2010).

The UPP functions by selecting ubiquitinated proteins for proteasomal degradation (Coux *et al.* 1996). The UPP contains the 26S proteasome, a large protein complex that is highly conserved in the cytosol and nucleus of eukaryotic organisms; the 26S proteasome is composed of a 20S proteolytic core that is bound by either one or two ATP-dependent 19S regulatory particles (Fujinami *et al.* 1994; Yang *et al.* 2004). Proteins delivered to the 26S proteasome are tagged with the small protein ubiquitin. Protein ubiquitination is dependent upon ubiquitin-activating enzymes (E1), ubiquitin-conjugating enzymes (E2) and ubiquitin ligases (E3) that transfer the ubiquitin onto the target protein (Smalle and Vierstra 2004). Proteolysis of ubiquitinated proteins in the 26S proteasome occurs in the 20S catalytic core. Moderate abiotic stressors can activate the UPP, which is associated with improved tolerance in plants (Lyzenga and Stone 2012). However, in mammalian cells it is well recognized that severe stress inhibits the UPP (Wang *et al.* 2010; Shang and Taylor 2011). In human cells, E1 and E2 enzymes are inhibited by superoxide accumulation (Jahngen-Hodge *et al.* 1997). Additionally, ROS can impair the 19S regulatory particles and promote 26S disassembly (Huang *et al.* 2013). In contrast to the 26S proteasome, the 20S proteasome contains the central 20S catalytic core, and functions by removing oxidized proteins in an ATP- and ubiquitin-independent manner (Davies 2001). Because the 20S proteasome lacks the 19S regulatory particles that are sensitive to oxidative stress, it is considered more resistant to oxidative stress compared with the 26S ubiquitin-dependent proteasome (Reinheckel *et al.* 1998; Kurepa *et al.* 2008).

The UPP governs nearly all facets of vascular plant development and physiology, including cell division (del Pozo *et al.* 2006), nutrient acquisition (Yates and Sadanandom 2013), hormone concentrations (Santner and Estelle 2010) and responses to a multitude of abiotic stressors, including heavy metals (Lyzenga and Stone 2012). The effect of heavy metals on proteasomal activity in higher plants is conflicting, which could reflect the different activities of the 20S and 26S proteasomes during oxidative stress, or concentration of the xenobiotic. For example, isolated 20S proteasomes from *Arabidopsis* had increased activity upon exposure to 50 μM cadmium (Polge *et al.* 2009), supporting the notion that the 20S proteasome is resistant to oxidative stress (Kurepa *et al.* 2008). However, total proteasome activity decreased in a crude extract from sunflower leaves exposed to 100–300 μM cadmium for 4 days (Pena *et al.* 2008). Similarly, proteasome activity decreased >2-fold in beans treated with 200 μM copper for 9 days (Karmous *et al.* 2014). Cadmium and copper are pro-oxidants that induce ROS, and the decrease in proteasome activity could reflect general impairment of the 26S proteasome caused by the concentration and long-term exposure of the heavy metals. Determining whether proteasomal activity in plants during heavy metal exposure is dose- and time-dependent warrants further study.

Although the UPP in higher plants is well characterized, it remains poorly understood in algae despite their ecological importance as primary producers. In the green alga *Chlamydomonas,* the UPP is known to have a role in flagella development (Huang *et al.* 2009) and the degradation of sulfate transporters (Pootakham *et al.* 2010), but its function in mitigating abiotic stress has not been fully investigated and only indirectly implicated. For example, chilling and heat stress increased the transcript abundance of ubiquitin in *Chlamydomonas* (von Kampen *et al.* 1995). Methyl viologen treatment generates superoxide accompanied with the accumulation of ubiquitinated proteins in *Chlamydomonas* (Shimogawara and Muto 1991); this likely suggests that the varied types of abiotic stress that induce superoxide would likewise increase the level of ubiquitinated proteins in *Chlamydomonas*. More recently, proteomic approaches have identified an accumulation of 20S core complex subunits in response to arsenate (Walliwalagedara *et al.* 2012) and cold stress in *Chlamydomonas* (Valledor *et al.* 2013). However, these previous studies did not determine whether the stressors increased proteasome activity, and therefore only partially implicate the UPP in mediating an abiotic stress response in *Chlamydomonas*.

This study characterized the involvement of the *Chlamydomonas* UPP in response to selenite stress. Selenium (Se) stress in plants is unique because it appears to have two distinct modes of toxicity (Van Hoewyk 2013). The speciation of available Se in soil solution and freshwater is predominantly selenate or selenite, both of which can act as pro-oxidants when transported into plants by depleting the glutathione pool (Van Hoewyk *et al*. 2008; Grant *et al.* 2011) and causing the accumulation of superoxide (Mroczek-Zdyrska and Wójcik 2012) and hydrogen peroxide in *Arabidopsis* (Lehotai *et al.* 2012) and the green algae *Ulva* sp. (Schiavon *et al.* 2012). In addition to Se inducing oxidative stress, it can also be toxic if it randomly replaces sulfur in proteins. This occurs when inorganic Se is assimilated into selenocysteine and selenomethionine, which can compete with tRNA^cys^ and tRNA^met^, respectively, and replace cysteine and methionine in proteins (Zhu *et al.* 2009). Given cysteine's role in catalysis and the formation of disulfide bonds that help stabilize protein structure, the replacement of cysteine by selenocysteine in proteins is likely to be particularly toxic (Stadtman 1990), because it can create malformed selenoproteins that potentially do not fold correctly. Therefore, Se stress can result in both oxidized proteins and malformed selenoproteins, both of which can induce a UPP response. The UPP was recently implicated in a Se response in *Stanleya pinnata* (Sabbagh and Van Hoewyk 2012)*,* a rare Se-hyperaccumulating plant that was able to selectively remove ubiquitinated selenoproteins. Whether or not the proteasome's ability to remove malformed selenoproteins represents a unique adaptation found exclusively in *S. pinnata* is not known.

In this study, the effect of moderate and severe oxidative stress on the UPP was investigated in selenite-treated *Chlamydomonas*, an algal species that is Se-sensitive and unicellular. Thus, it was feasible to determine how the concentration of Se affected both ROS production and the UPP on a cellular level. The data demonstrate that the UPP protected *Chlamydomonas* against the toxic effects of Se, and could alleviate Se toxicity by removing malformed selenoproteins. However, proteasome activity and the accumulation of ubiquitinated proteins in response to Se treatment were both time and dose dependent, and were associated with levels of ROS. To our knowledge, this study is the first to measure proteasome activity in *Chlamydomonas*, and provides a more comprehensive understanding of the effects of ROS accumulation on the UPP in lower plants.

## Methods

### Growth conditions

*Chlamydomonas reinhardtii* (wild-type strain CC-1690) was obtained from the *Chlamydomonas* Resource Center (University of Minnesota, MN, USA) and cultured axenically with or without sodium selenite in tris-acetate-phosphate (TAP) media under constant illumination (100 μE) and shaking (150 rpm) at 24 °C. Cultures were initially inoculated in 50 mL of sterile TAP media containing 1000 cells mL^−1^; cell counts were performed with a haemocytometer. During this time, the pH was checked and adjusted if necessary to pH 7–7.2.

Chlorophyll from 1 mL of culture was extracted in *N*,*N*-dimethylformamide and measured spectrophotometrically at an absorbance of 652 nm and subtracted for turbidity at 750 nm (Arnon 1949). The effect of proteasome inhibition in cells treated with 0, 50 and 200 μM was determined by measuring chlorophyll content as described above, except that 5 mL cultures were treated with either 0.1 % (v/v) dimethyl sulfoxide (DMSO) (control) or 10 μM of MG132 dissolved in 0.1 % DMSO; the chlorophyll content was measured every 24 h for 96 h to gauge the effect of proteasome inhibition during growth at logarithmic and stationary phases.

### Protein electrophoresis

To estimate the effects of selenite on protein oxidation, cells were grown to an OD_0.5_ in TAP media with or without sodium selenite. Cells were harvested (50 mL) by centrifugation at 1500 *g* at various time points (0, 3, 8 and 28 h). Proteins were extracted in a protein extraction buffer (100 mM NaCl, 50 mM Tris, pH 7.5, 0.5 % (v/v) TritonX-100, 1 mM dithiothreitol and 1 mM phenylmethanesulfonylfluoride) using three repeated freeze–thaw cycles. Protein concentrations were determined as described (Bradford 1976). Oxidized proteins were detected using the OxyBlot Protein Oxidation Detection Kit (Millipore Company). Briefly, 10 μg of protein were derivatized with 2,4-dinitrophenylhydrazine (DNP) and separated by 10 % sodium dodecyl sulfate–polyacrylamide gel electrophoresis. Proteins were transferred onto PVDF membranes by electroblotting and detected with anti-DNP primary antibody. The immunoreactive proteins were detected using an anti-rabbit secondary antibody conjugated to alkaline phosphatase. For comparison, and to ensure equal loading, 10 μg of protein were separated on another 10 % SDS gel and the large subunit of Rubisco was detected with antiserum raised in rabbits (Agrisera).

The accumulation of ubiquitinated proteins was determined in cells treated with 0, 50 or 200 μM selenite for various lengths of time (0, 3, 8 and 28h) in the presence of 10 μM MG132 in 0.1 % DMSO; this time course was selected because it was previously demonstrated that levels of ubiquitinated proteins can rapidly accumulate in cultures of *Arabidopsis* cells challenged with oxidative stress (Nishizawa-Yokoi *et al.* 2010). Briefly, 50 μg of protein (extracted as stated above) were separated on an 8 % SDS gel; high-molecular-weight poly-ubiquitinated proteins were detected using ubiquitin antiserum raised in mouse as previously described (Sabbagh and Van Hoewyk 2012). On a separate gel, 20 μg of protein were separated on a 10 % SDS gel and stained with Coomassie blue to ensure equal loading of proteins.

To determine the intactness of the 20S and 26S proteasomes, *Arabidopsis* plants were grown on agar plates containing Hoagland's media for 10 days before being transferred to sterile flasks containing 50 mL of Hoagland's media. After 24 h of constant shaking, plants were treated with or without 50 μM selenite and sampled at various time points (0, 8, 24 and 72 h). Leaves were ground in liquid nitrogen and non-denatured proteins were extracted in a proteasome extraction buffer (50 mM potassium-phosphate buffer—pH 7.4, 5 % (v/v) glycerol, 10 mM ATP, 5 mM β-mercaptoethanol). Non-denatured proteins (50 μg) were separated for 4 h at 4 °C on a 6 % non-denaturing gel containing 2 mM ATP to ensure the intactness of the ATP-dependent 26S proteasome (Yang *et al.* 2004). The Pba1 antiserum (Santa Cruz Biotechnology) used in this study reacted against the 20S catalytic core in *Arabidopsis*, but not *Chlamydomonas*. Immunoreactive proteins transferred onto PVDF were detected as described above.

### Proteasome activity

*Chlamydomonas* in an exponential phase (OD_0.5_) were cultured and then treated with or without 50 and 200 μM sodium selenite. Proteins from harvested cells treated for 0, 3, 8 and 28 h were extracted under non-denaturing conditions in a proteasome extraction buffer using repeated freeze–thaw cycles. Protein extract concentrations were determined using the Bradford method. The chymotrypsin activity of the proteasome was measured essentially as described (Yang *et al.* 2004). Briefly, the activity from three separate cultures was measured fluorometrically in a 96-well plate (Ex_360_/Em_410_) containing 10 μL of protein extract and 90 μL of reaction buffer (50 mM potassium-phosphate buffer, 2 mM MgCl_2_, 1 mM ATP, 5 mM β-mercaptoethanol) with 50 μM of the fluorogenic peptide Suc-LLVY-AMC dissolved in DMSO. The released fluorescence signals were recorded every 5 min under PHERA star (BMG Labtech). For each time point (cell culture treated with or without Se for 0, 3, 8 and 28 h), proteasome activity was determined after 1 h as the difference in increased fluorescence in reactions with or without MG132 (50 μM) to account for non-proteasomal release of AMC. Therefore, the proteasome activity is expressed as the net changes of fluorescence (RFU min^−1^ μg protein^−1^) in the presence and absence of the proteasome inhibitor MG132. The proteasome activity in Se-treated cultures was expressed as fold change relative to untreated cultures at each time point.

### Elemental analysis of proteins

In order to maximize biomass, cells were grown to an OD_0.8_ in 800 mL of TAP media (*n* = 4 separate cultures) containing either 50 or 200 µM sodium selenite with or without 10 μM MG132 in 0.1 % DMSO for 8 h, and then harvested by centrifugation at 1500 *g*. Proteins were extracted as described above and extract concentrations were determined using the Bradford method. Proteins were precipitated with trichloroacetic acid (TCA, final concentration 10 %), as previously described (Grant *et al.* 2011). Protein pellets were digested in 0.5 mL of nitric acid at 95 °C overnight, and brought to 5 mL using 18 mega Ohm DI water. Samples were then filtered (0.45 μm) and analysed using inductively coupled plasma-mass spectroscopy (ICP-MS) at North Carolina State University, USA.

### ROS measurements and microscopy

The cell permeable fluorescent probe 2′,7′-dichlorodihydrofluorescein diacetate (H_2_DCFDA) was used to visualize ROS in *Chlamydomonas*. Additionally, the probe MitoSox Red (Molecular Probes, Invitrogen) that selectively fluoresces in the presence of mitochondrial superoxide was used in separate experiments to determine if Se toxicity induces mitochondrial superoxide. A 3 mL culture of algae (OD_0.5_) treated with or without 50 μM selenite for 0, 1, 3, 8 and 28 h was harvested by centrifugation at 1500 *g* and resuspended in 1 mL of TAP media containing 10 μM H_2_DCFDA or 5 μM of MitoSox Red dissolved in DMSO; the 1-h time-point was added to determine if Se rapidly induces ROS. Cells were incubated on a rotating platform in the dark for 20 min and then washed three times with TAP media to remove the residual H_2_DCFDA or MitoSox Red to avoid background fluorescence. The relative fluorescence units of H_2_DCFDA_(ex 492/em 525)_ and Mitosox_(ex 510/em 580)_ were determined using a FITC and TRITC filter set, respectively, on a spectrofluorometer. The fluorescent values were corrected by determining the autofluorescence of samples without H_2_DCFDA or MitiSox. Localization of H_2_DCFDA and Mitosox was visualized using an Olympus FV1200 confocal laser microscope. Autofluorescence of *Chlamydomonas*' single cup-shaped chloroplast was artificially depicted as blue fluorescence to distinguish it from the red fluorescence of MitoSox. Fluorescence of H_2_DCFDA and MitoSox Red was determined at Ex_492_/Em_525_ and Ex_510_/Em_580_, respectively.

Cell viability was determined by estimating the intactness of the cell membrane by using the fluorescent probe fluorescein diacetate (FDA) according to Prado *et al.* (2011). Briefly, cells treated with or without selenite were incubated with FDA for 15 min in the dark and then washed three times with TAP media. Viable cells with intact membranes retained the fluorescent probe and were visualized using a FITC filter set. The percentage of viable cells was estimated by dividing the number of fluorescent cells by the number of total cells viewed under visible light.

### Statistical analysis

For measuring cell viability, proteasome activity and chlorophyll contents in cells treated with or without selenite, a parametric one-way analysis of variance (ANOVA) was used. If ANOVA analysis was significant (*P* < 0.05), multiple comparisons were made using Tukey's *post-hoc* test. The effect of proteasome inhibition on the elemental composition of proteins was determined by Student's *t*-test at the 0.05 probability level. Values are expressed as mean ± SEM. Both ANOVA and Student's *t*-test as well as data plotting were performed using the KaleidaGraph software program (Synergy Software).

## Results

### The effects of Se on the UPP

In preliminary experiments*, C. reinhardtii* were grown in TAP media and supplemented with 0, 10, 50, 100 and 200 μM selenite for 96 h. The toxicity of selenite was dose dependent in *Chlamydomonas* **[see Supporting Information]**, as previously reported (Morlon *et al.* 2005). Two selenite concentrations of 50 μM, which is just below the reported IC_50_, and 200 μM were used in subsequent experiments to investigate how moderate and severe selenite stress affects the UPP.

**Figure 1.**
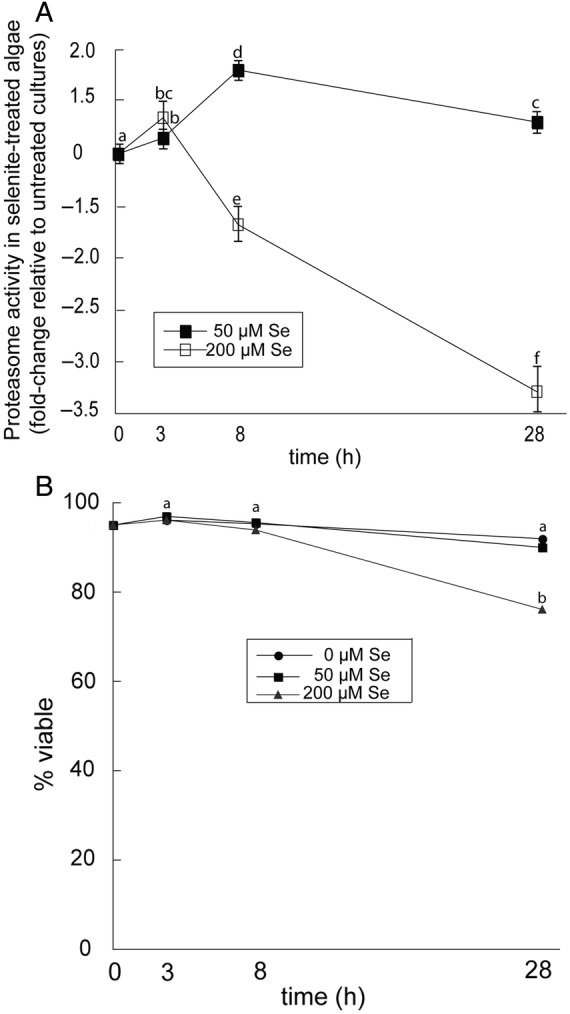
Proteasome activity in *Chlamydomonas* treated with selenite. (A) Values for proteasome activity represent fold change in activity of selenite-treated cells relative to untreated cells at each time interval. (B) Percentage of viable cells in cultures with the indicated treatments. Data are the mean of three biological replicates and standard deviation. Lowercase letters represent a significant difference in relative activity at each time point (*P* < 0.05). Standard errors were too small to plot.

To determine whether or not moderate and severe selenite stress induces the UPP, *Chlamydomonas* cells were grown to an OD_0.5_ and treated with or without 50 and 200 μM selenite. Compared with untreated cells, 50 μM selenite increased proteasome activity after 3, 8 and 28 h (Fig. 1A). Proteasome activity peaked in cells treated with selenite after 8 h, at which point activity was 1.8-fold higher compared with untreated cells. Curiously, however, the proteasome activity in 50 μM selenite-treated cells decreased at 28 h compared with 8 h, but activity was still 1.3-fold higher compared with untreated cells at the same time point. Severe Se stress caused by 200 μM selenite also increased proteasome activity after 3 h, but decreased 1.5- and 3-fold after 8 and 28 h, respectively, compared with untreated cells at the same time point. Cell viability was investigated to determine if the decreased proteasome activity caused by 200 μM selenite was a consequence of cell death. There was no difference in cell viability among the different treatments at 3 and 8 h (Fig. 1B). Compared with untreated cells at 28 h, viability decreased from 92 to 76% in cultures treated with 200 μM selenite. However, this decrease in viability does not coincide with the 3-fold decrease in proteasome activity after 28 h.

**Figure 2.**
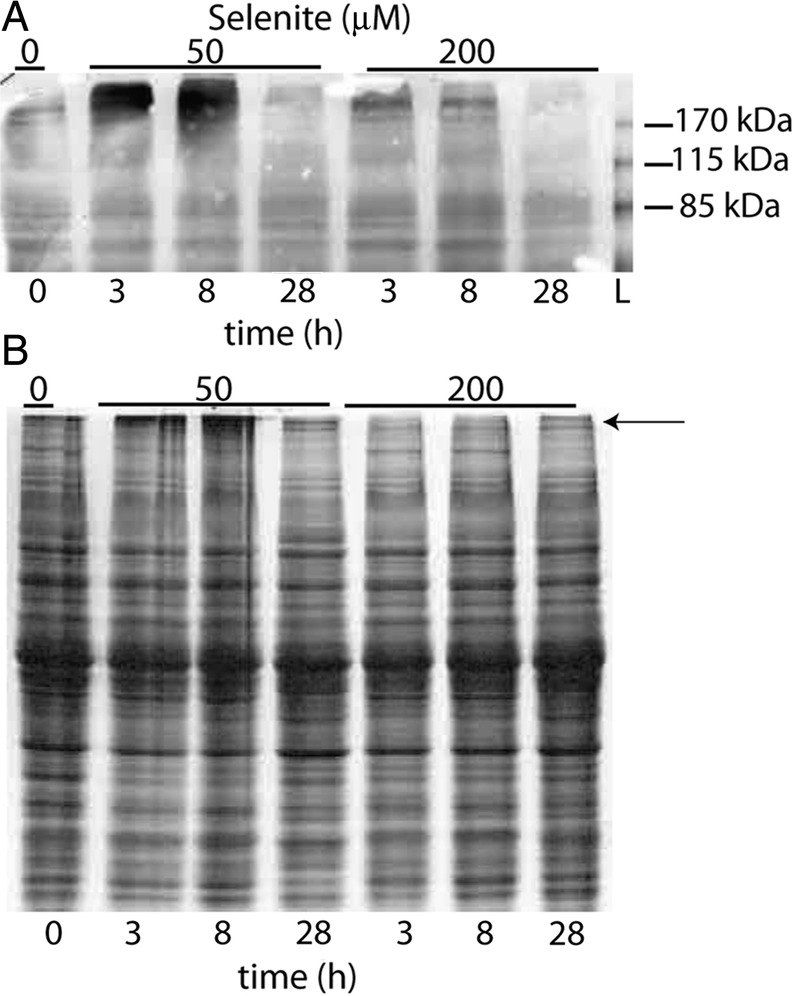
The accumulation of high-molecular-weight ubiquitinated proteins in *Chlamydomonas* treated with or without selenite at different time points. (A) 50 μg of protein were separated on an 8 % SDS gel and ubiquitinated proteins were detected using anti-ubiquitin antiserum. The immunoblot is representative of two other replicate gels. (B) 20 μg of protein were separated on a 10 % SDS gel and stained with Coomassie to ensure equal loading between lanes. The arrow points to suspected high-molecular-weight ubiquitinated proteins. L, ladder.

As noted above, proteasome activity during Se treatment was both time and dose dependent. We deemed it worthwhile to determine if levels of ubiquitinated proteins were also affected by duration and concentration of Se treatment. During moderate stress induced by 50 μM selenite, high-molecular-weight ubiquitinated proteins were most abundant after 3 and 8 h, but decreased after 28 h of Se-treatment (Fig. 2). Compared with 50 μM selenite, accumulation of ubiquitinated proteins notably declined during severe stress caused by 200 μM selenite after 3 and 8 h, and were absent at 28 h. Coomassie staining of a separate gel confirmed that there was not a significant difference in protein banding between the samples, except for the appearance of high-molecular weight proteins near the top of the gel, presumed to be ubiquitinated proteins (Fig. 2). Together, these data indicate that the accumulation of ubiquitinated proteins induced by selenite is both time and dose dependent, perhaps suggesting that the severity of selenite-induced oxidative stress impairs the ubiquitination of substrate proteins.

### The UPP removal of malformed selenoproteins

**Figure 3.**
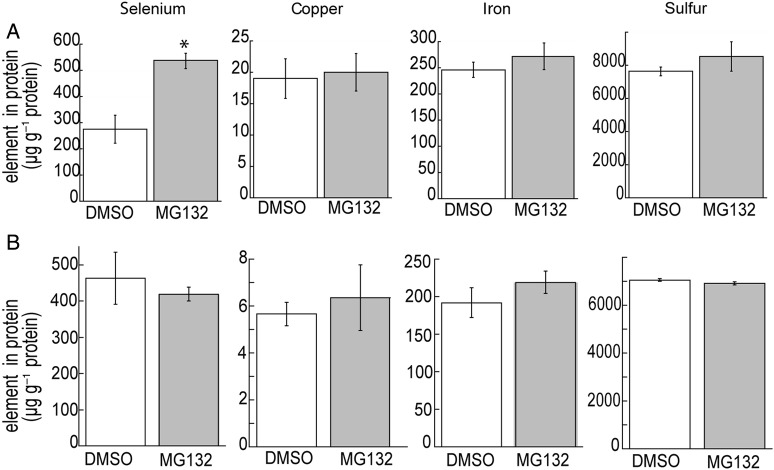
The effect of proteasome inhibition on the elemental composition of proteins. The concentrations of Se, copper, iron and sulfur in proteins from cells treated with 50 μM selenite (A) and 200 μM selenite (B) were analysed. Proteins were precipitated from *Chlamydomonas* after 8 h of Se treatment with or without MG132, and then analysed by ICP-MS. Shown are the mean and standard error (*n* = 4 protein samples from separately grown cultures). The asterisk above the scale bars represents a significant difference between treatments (*P* < 0.05).

Selenite can be assimilated into organic seleno-amino acids in land plants (Zhu *et al.* 2009) and green algae, including *Scenedesmus* (Umysová *et al.* 2009). The malformed selenoprotein hypothesis predicts that the random replacement of cysteine with selenocysteine produces diselenide bonds that potentially results in aberrant and misfolded selenoproteins (Brown and Shrift 1982; Van Hoewyk 2013). In support of this theory, treatment with selenocysteine increased both proteasome activity and levels of ubiquitinated proteins **[see Supporting Information]**. Although proteasomal removal of selenoproteins is facilitated by ubiquitin (Sabbagh and Van Hoewyk 2012), we reasoned that because 200 μM selenite decreased the abundance of ubiquitinated proteins, the impaired UPP might not be able to remove malformed selenoproteins for proteolysis. To determine if the proteasome in *Chlamydomonas* has the capacity to potentially remove non-specific selenoproteins, the elemental content of proteins was determined from cells treated with 50 and 200 μM selenite for 8 h in the presence or absence of the proteasome inhibitor MG132. If the proteasome functions to remove malformed selenoproteins, then inhibition of the proteasome would be expected to increase the amount of Se in proteins. Proteasome inhibition in cells treated with 50 μM selenite increased the concentration of Se in protein 2-fold compared with control cells without MG132 (Fig. 3A), indicating that a functioning UPP removes malformed selenoproteins. However, the concentrations of copper, iron and sulfur did not differ in cells treated with MG132 or DMSO. As expected, Se in protein increased in cells treated with 200 μM selenite compared with 50 μM selenite. However, at this selenite concentration, levels of Se in protein were not affected by MG132 (Fig. 3B). This suggests that the proteasome's ability to remove malformed selenoproteins is dose-dependent, and it is impaired during severe Se stress. This is consistent with the observation that ubiquitinated proteins do not accumulate at 200 μM selenite, as they did at 50 μM selenite.

### UPP protects *Chlamydomonas* from Se stress

**Figure 4.**
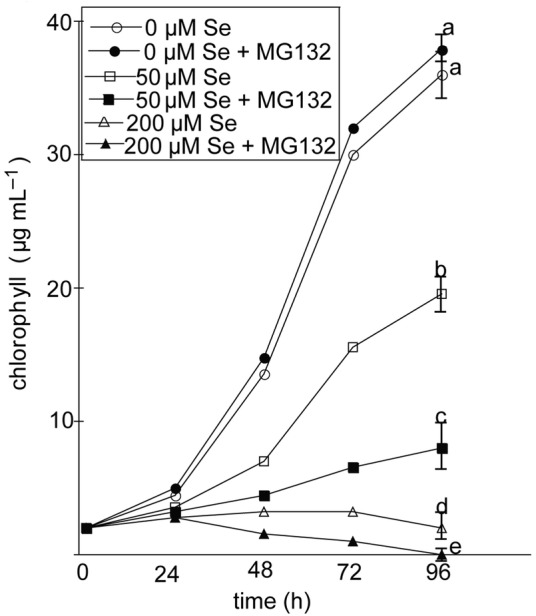
The effects of proteasome inhibition on selenite toxicity. Algae were inoculated with 1000 cells mL^−1^ and cultured for 96 h containing 0, 50 and 200 μM selenite with or without MG132. Chlorophyll content was analysed every 24 h. Data shown are the mean (*n* = 4 separate cultures). Lowercase letters represent significant difference at 96 h (*P* < 0.05).

The role of the proteasome in alleviating selenite toxicity was examined by treating cells with or without MG132 for 96 h. Proteasome inhibition did not affect the chlorophyll content in cells cultured without selenite. In contrast, the chlorophyll content of cells grown with 50 and 200 μM selenite decreased in cells treated with MG132 (Fig. 4), indicating that the functioning proteasome protects *Chlamydomonas* against Se stress. Notably, the protective benefits of the proteasome during Se stress was more apparent at 50 μM compared with 200 μM selenite, which is likely due to UPP impairment at the higher selenite concentration.

### Selenite-induced ROS production

As elaborated above, the UPP response during Se stress is both dose and time dependent. We deemed it is necessary to determine whether the decreased proteasome activity and ubiquitinated proteins during severe Se stress were a consequence of the proteasome alleviating and removing the Se-induced stress, or rather if severe Se stress might impair the UPP. Since severe oxidative stress can impair 26S proteasome activity (Shang and Taylor 2011), it was desirable to determine whether selenite-treated *Chlamydomonas* exhibited signs of oxidative stress, which could possibly explain why proteasome activity decreased during severe Se stress. Reactive oxygen species are produced primarily in the mitochondria and chloroplast during stress; recently, it was reported that cadmium-treated *Arabidopsis* accumulate ROS in mitochondria prior to being observed in plastids (Bi *et al.* 2009). Therefore, the fluorescent probes 2′,7′-dichlorofluorescein diacetate (H_2_DCFDA) and MitoSox were used to determine the accumulation of ROS in selenite-treated cells. H_2_DCFDA fluorescence is not specific to the type of ROS or cellular compartment whereas MitoSox specifically fluoresces in the presence of mitochondrial superoxide.

**Figure 5.**
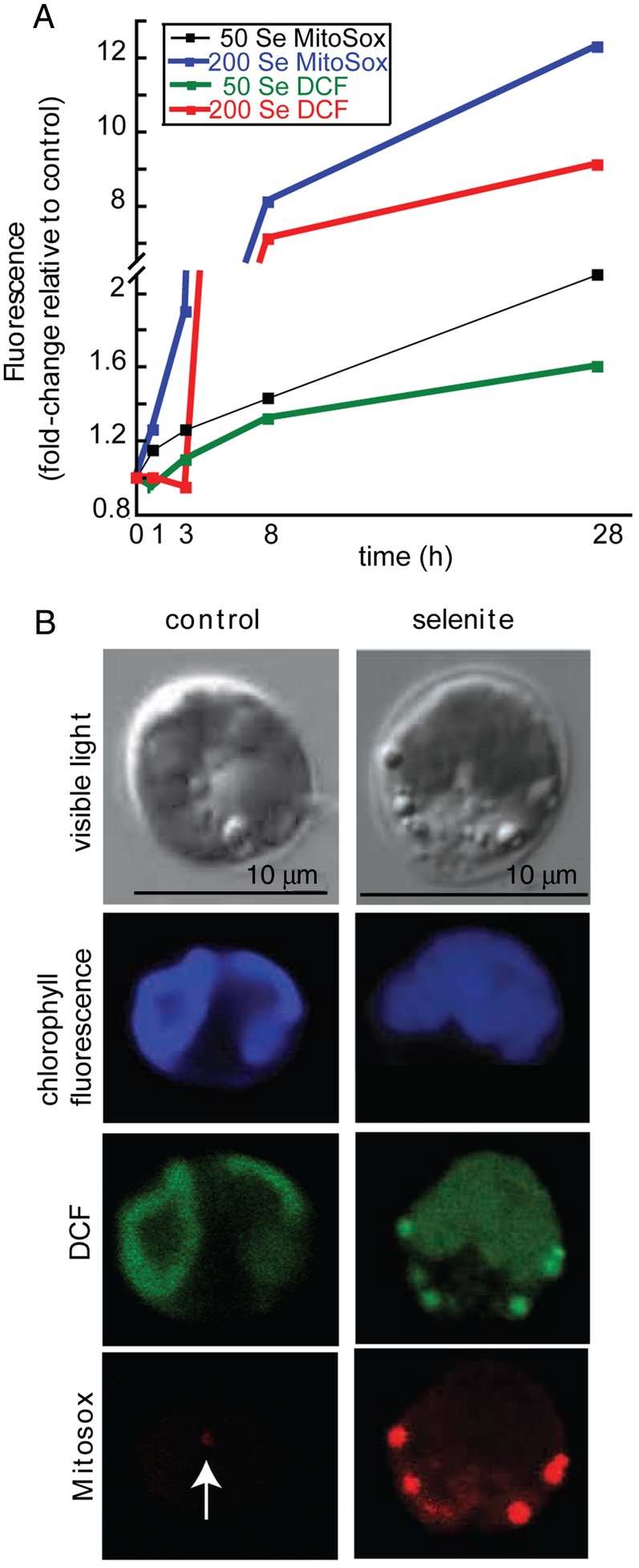
The effect of selenite on the accumulation of ROS in *Chlamydomonas*. (A) Values represent relative fold change of H_2_DCFDA and MitoSox fluorescence compared with untreated cells at each time point and are corrected for autofluorescence. Shown are the mean (*n* = 5 separate cultures) at 0, 3, 8 and 28 h. (B) Confocal microscopy reveals DCF and MitoSox fluorescence in cells untreated and treated with 200 μM selenite for 8 h, and was used to confirm that MitoSox was not localized to the chloroplast or cytosol. Arrow points to MitoSox fluorescence in the untreated cell.

The results showed that fluorescence of H_2_DCFDA and MitoSox increased in Se-treated cells in a time- and dose-dependent manner (Fig. 5A). After treating cells with 50 μM selenite for 28 h, H_2_DCFDA and MitoSox fluorescence increased 1.6- and 2.1-fold (fluorescence of treated/untreated cells) respectively. Treatment with 200 μM selenite increased H_2_DCFDA and MitoSox fluorescence 9- and 12-fold, respectively, compared with untreated cells. Collectively, these data indicate that selenite continuously induced the formation of ROS, and in particular mitochondrial superoxide, which leads us to believe that the decreased proteasome activity was not a consequence of alleviating the Se-induced stress.

The accumulation of mitochondrial superoxide as determined by MitoSox has not been previously reported in *Chlamydomonas* as it has for other organisms including *Arabidopsis* (Cvetkovska and Vanlerberghe 2012). Thus, it was essential to rule out that the MitoSox was not localized to the chloroplast or diffused into the cytosol. Confocal microscopy was used to gauge the localization of MitoSox in cells treated with 200 μM selenite for 8 h (Fig. 5B). The results showed that while H_2_DCFDA fluorescence was visible in the chloroplasts of both control and treated cells, it was also only apparent in bright regions outside the chloroplast in Se-treated cells. MitoSox fluorescence appeared predominantly in Se-treated cells. Although it was not possible to confirm that MitoSox was indeed strictly localized to the mitochondria, the current observation suggests that the fluorescent probe did not diffuse into the cytosol or cross the chloroplastic membranes.

**Figure 6.**
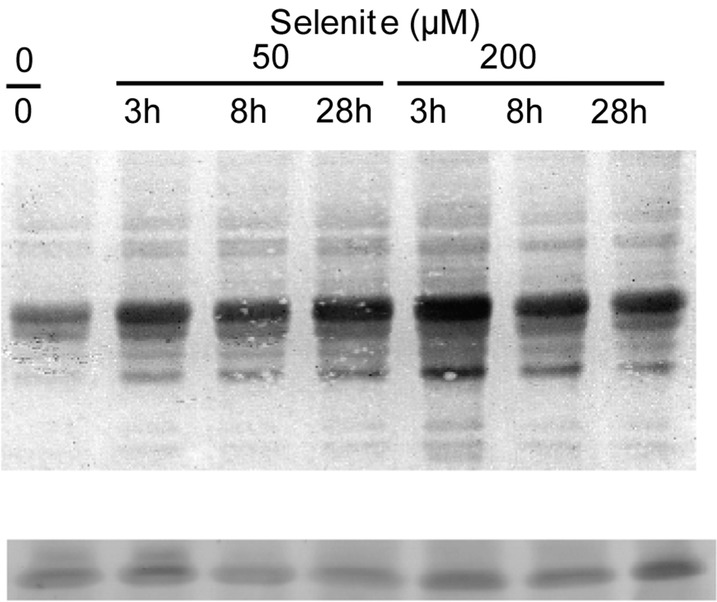
The effect of selenite on oxidized proteins in *Chlamydomonas*. 10 μg of protein were derivatized with 2,4-DNP and separated on a 10 % SDS gel. Oxidized proteins were detected with DNP antibodies. The immunoblot is representative of two other gels. Shown below on a separate gel are levels of the large subunit of Rubisco from 20 μg of protein separated on a 10 % SDS gel.

Given that selenite-treated *Chlamydomonas* accumulated ROS, we sought to determine if Se increased the amount of oxidized proteins, which potentially could be delivered to the proteasome for removal. Compared with untreated cells, selenite induced the formation of oxidized proteins at all time points in cells treated with 50 and 200 μM selenite (Fig. 6). However, the accumulation of the oxidized proteins was not associated with levels of Se-induced ROS. For example, at 200 μM selenite, levels of oxidized proteins were highest after 3 h, even though ROS were most abundant at 28 h.

Severe oxidative stress in yeast and human cells can also result in the disassembly of the 19S regulatory particle from the 20S catalytic core (Wang *et al.* 2010; Huang *et al.* 2013). The intactness and accumulation of the 20S and 26S proteasomes during prolonged Se-treatment were investigated using an antibody that reacts against the *Arabidopsis* Pba1, a subunit of the 20S core particle. However, the antibody did not react against the *Chlamydomonas* proteasome. Yet in *Arabidopsis*, the abundance of the 26S proteasome decreased 3 days after Se-treatment [**see Supporting Information****]**.

## Discussion

### The UPP protects algae from Se toxicity

Photosynthetic organisms display a wide range of tolerance to Se. The UPP in *S. pinnata* was recently implicated in a Se-stress response (Sabbagh and Van Hoewyk 2012). The Se-tolerant plant *S. pinnata* has a preference for Se, and has evolved mechanisms that allow it to accumulate Se without the detrimental effects that would be observed in most plants (Freeman *et al.* 2010). Thus, the disparity between *S. pinnata* and *Chlamydomonas* cannot be overlooked. *Chlamydomonas*, which is at the other end of the spectrum of Se tolerance compared with *S. pinnata*, is now also known to invoke the UPP during moderate Se stress.

Using both molecular and physiological approaches, four lines of evidence suggest that the UPP is implicated when *Chlamydomonas* were treated with 50 μM selenite. First, proteasome activity increased nearly 2-fold after 8 h of Se-treatment in *Chlamydomonas*. The increased proteasomal activity was also associated with an accumulation of ubiquitinated protein in cells treated with Se for 3 and 8 h. Additionally, inhibition of the proteasome increased the amount of Se in protein during moderate Se stress, suggesting that a functional UPP acts to remove malformed and misfolded selenoproteins. Lastly, proteasome inhibition decreased the chlorophyll content of selenite-treated cells after 96 h, indicating that the proteasome protects *Chlamydomonas* from the toxic effects of selenite. Inhibition of the proteasome during Se stress proved to be more toxic to *Chlamydomonas* due to the inefficient removal of damaged proteins, including malformed selenoproteins, which likely led to an accumulation of protein aggregates in the cytosol. Thus, the protective benefits of a UPP during Se stress in *Chlamydomonas* likely extend to other types of plants, possibly including crops.

Although it remains to be demonstrated, it is proposed that the UPP can function by removing non-specific selenoproteins in all domains of life, including *Chlamydomonas*, humans and other organisms that require Se to make specific and essential selenoproteins (Novoselov *et al.* 2002). In support of this argument, recently it was shown that human cells treated with selenocystine display an unfolded protein response, including increased protein ubiquitination and ER stress (Wallenberg *et al.* 2014). Similarly, *Chlamydomonas* treated with selenocystine also have increased levels of ubiquitinated proteins and proteasome activity.

### Mitochondrial superoxide impairs the UPP in algae

Although moderate stress caused by 50 μM selenite was associated with increased UPP involvement, severe oxidative stress impaired the UPP. At a concentration of 200 μM selenite, an increased accumulation of ROS was associated with a concomitant decrease in proteasomal activity. This study demonstrated that Se induces mitochondrial superoxide, which may impair the UPP in lower plants. In mammalian cells, selenite also induces mitochondrial superoxide (Wallenberg *et al.* 2010). A recent study in human cells noted that mitochondrial superoxide impaired stability and activity of the 26S proteasome (Huang *et al.* 2013). It is likely that the assembly of the 26S proteasome in *Chlamydomonas* is also impaired during severe or prolonged Se exposure, but this could not be experimentally determined as it was for *Arabidopsis*. The Rpn2 (Zmijewski *et al.* 2009) and S6 ATPase subunits of the 19S regulatory particle (Ishii *et al.* 2005) are among the most easily oxidized proteins of the 26S proteasome and damage to these subunits is accompanied by decreased proteasome activity. It is possible that mitochondrial superoxide similarly oxidized subunits of the 19S regulatory particle in *Chlamydomonas*, which led to the observed decrease in proteasome activity at an increased selenite concentration or exposure. Alternatively, because ATP depletion has been shown to affect 26S proteasome stability in *Arabidopsis* (Yang *et al.* 2004), it is also feasible that impairment of UPP in *Chlamydomonas* could be explained by a decrease in ATP levels caused by altered mitochondrial processes that were affected by mitochondrial superoxide; however, this remains to be experimentally demonstrated in *Chlamydomonas*.

Inhibition of the UPP is also manifested by the absence of ubiquitinated proteins in cells treated with 200 μM selenite, as well as in cells treated with 50 μM selenite for 28 h compared with 3 and 8 h. The apparent dose- and time-dependent effects of selenite on the accumulation of ubiquitinated proteins in *Chlamydomonas* are strikingly similar to a study using human cells treated with cadmium; Figueiredo-Pereira *et al.* (1998) report that the accumulation of ubiquitinated proteins decreased if cells were treated with increasing concentrations of cadmium or exposed to the heavy metal for longer periods of time. Severe oxidative stress, including mitochondrial superoxide generation, can impair the UPP by directly inhibiting mammalian E1 ubiquitin-activating and E2-conjugating enzymes (Jahngen-Hodge *et al.* 1997; Huang *et al.* 2013). The observed absence of ubiquitinated proteins during severe Se-induced oxidative stress (Fig. 2) might similarly be caused by E1 and E2 impairment, although this hypothesis remains to be experimentally validated in plants. Additionally, compared with 50 μM selenite, proteasome inhibition in cells treated with a 200 μM selenite did not increase the amount of Se in proteins (Fig. 3B). The proteasomal removal of malformed selenoproteins is likely to be dependent upon ubiquitin (Sabbagh and Van Hoewyk 2012), which implicates the 26S proteasome. During severe Se stress, the decreased ability to ubiquitinate proteins likely prevented the removal of malformed selenoproteins, supporting the hypothesis that the UPP was damaged.

## Conclusions

The role of the UPP in mediating an abiotic stress response in higher plants is well characterized, but is less defined in algae. The aim of this study is to better understand the short-term effects of selenite-induced oxidative stress on the UPP. The data indicate that mild selenite stress in *Chlamydomonas* invokes the UPP, and to our knowledge, this is the first study to report increased proteasomal activity in an algal species in response to stress. In contrast to mild stress, severe Se stress impaired the UPP, which was associated with ROS accumulation, including mitochondrial superoxide. Therefore, this study provided the insight that the UPP in plants is affected by ROS, which were shown to increase in a time- and dose-dependent manner during Se treatment. Clearly the mounting evidence indicates the importance of the UPP in plant stress physiology. However, because the UPP is sensitive to oxidative stress, the extent of an organism's capacity to quench ROS likely dictates UPP activity and its role in abating stress.

## Sources of Funding

This work was supported by the United States National Science Foundation (MCB-1244009 awarded to D.V.H.).

## Contributions by Authors

C.-Y.H., J.X. and D.V.H. conceived and designed the experiments. P.V., C.-Y.H. and D.V.H. performed the experiments and analysed the data. D.V.H. created the figures. D.V.H., C.-Y.H. and J.X. wrote and prepared the manuscript.

## Conflicts of Interest Statement

None declared.

## Supporting Information

The following Supporting Information is available in the online version of this article –

**Figure S1.** The effect of selenite on cell density.

**Figure S2.** The effect of selenocystine on the UPP.

**Figure S3.** The effect of selenite on the abundance of the *Arabidopsis* 20S and 26S proteasomes.

Additional Information
