# Supplementary material for: The ubiquitin–proteasome pathway protects Chlamydomonas reinhardtii against selenite toxicity, but is impaired as reactive oxygen species accumulate
Source: AoB Plants. 2014 Oct 8;6:plu062. doi: 10.1093/aobpla/plu062 (PMC4231294; doi:10.1093/aobpla/plu062)
Supplement: Additional Information [file supp_6_plu062_index.html]

The ubiquitin-proteasome pathway protects Chlamydomonas reinhardti against selenite toxicity, but is impaired as reactive oxygen species accumulate — The ubiquitin–proteasome pathway protects Chlamydomonas reinhardtii against selenite toxicity, but is impaired as reactive oxygen species accumulate — Additional Information 

# The ubiquitin–proteasome pathway protects *Chlamydomonas reinhardtii* against selenite toxicity, but is impaired as reactive oxygen species accumulate

## Additional Information

Additional Information

**Files in this Data Supplement:**

- Supporting Information - docx file
