# Supplementary material for: The ubiquitin–proteasome pathway protects Chlamydomonas reinhardtii against selenite toxicity, but is impaired as reactive oxygen species accumulate
Source: AoB Plants. 2014 Oct 8;6:plu062. doi: 10.1093/aobpla/plu062 (PMC4231294; doi:10.1093/aobpla/plu062)
Supplement: Additional Information [file supp_plu062_plu062supp.docx]

Figure S1. The effect of selenite on cell density. The cell density of *Chlamydomonas* cultures treated with 0, 10, 50, and 200 μM selenite were determined 96 hours after inoculating cells (1000 cell ml^-1^) in 50 mL of sterile TAP media. Data shown are the mean (n=4 separate cultures) and standard error. Lowercase letters represent significant difference (*p* < 0.05).

Figure S2. The effect of selenocystine on the UPP. (a) Proteasome activity in *Chlamydomonas* treated with 6 μM selenocystine after 3 and 8 h. Values represent fold-change in activity of selenocystine-treated cells relative to untreated cells at the each time interval. Data are the mean (n=3 separate cultures) and standard error. Lowercase letters represent significant difference (*p*< 0.05) compared to untreated cultures. (b) The accumulation of high-molecular weight ubiquitinated proteins in *Chlamydomonas* treated with or without selenocystine at different time points. 50 μg of protein were separated on an 8% SDS gel, and ubiquitinated proteins were detected using anti-ubiquitin antiserum. The immunoblot is representative of one other replicate gel. L = ladder.

Figure S3. The effect of selenite (50 μM) treatment on the abundance of the Arabidopsis 20S and 26S proteasome containing one or two regulatory lids at different time intervals. Intact complexes of the proteasomes were determined by separating 50 μg of protein on a 6% non-denaturing gel containing ATP, and immunoblotted with the Pba1 antiserum that reacts against a subunit of the 20S proteolytic core (top). Shown on the bottom are levels of the Pba1 protein detected on 12% SDS-PAGE containing 20 μg of denatured protein per lane.
